# International Variability of Barriers to Adherence to Immunosuppressive Medication in Adult Heart Transplant Recipients. A Secondary Data Analysis of the BRIGHT Study

**DOI:** 10.3389/ti.2024.12874

**Published:** 2024-08-29

**Authors:** Kris Denhaerynck, Gabriele Berger Wermuth, Fabienne Dobbels, Lut Berben, Cynthia L. Russell, Sabina De Geest

**Affiliations:** ^1^ Department of Public Health, Institute of Nursing Science, University of Basel, Basel, Switzerland; ^2^ Department of Public Health and Primary Care, Academic Centre for Nursing and Midwifery, KU Leuven, Leuven, Belgium; ^3^ School of Nursing and Health Studies, University of Missouri-Kansas City, Kansas, MO, United States

**Keywords:** heart transplantation, medication adherence, immunosuppressant nonadherence, immunosuppressant medication, barrier

## Abstract

Non-adherence to immunosuppressive medication among transplant patients is associated with poor clinical outcomes and higher economic costs. Barriers to immunosuppressives are a proximal determinant of non-adherence. So far, international variability of barriers to adherence in transplantation has not been studied. As part of the cross-sectional multi-country and multi-center BRIGHT study, barriers to adherence were measured in 1,382 adult heart transplant recipients of 11 countries using the 28-item self-report questionnaire “Identifying Medication Adherence Barriers” (IMAB). Barriers were ranked by their frequency of occurrence for the total sample and by country. Countries were also ranked the by recipients’ total number of barriers. Intra-class correlations were calculated at country and center level. The five most frequently mentioned barriers were sleepiness (27.1%), being away from home (25.2%), forgetfulness (24.5%), interruptions to daily routine (23.6%) and being busy (22.8%), fairly consistently across countries. The participants reported on average three barriers, ranging from zero up to 22 barriers. The majority of the variability among reported barriers frequency was situated at the recipient level (94.8%). We found limited international variability in primarily person-level barriers in our study. Understanding of barriers in variable contexts guides intervention development to support adherence to the immunosuppressive regimen in real-world settings.

## Graphical Abstract

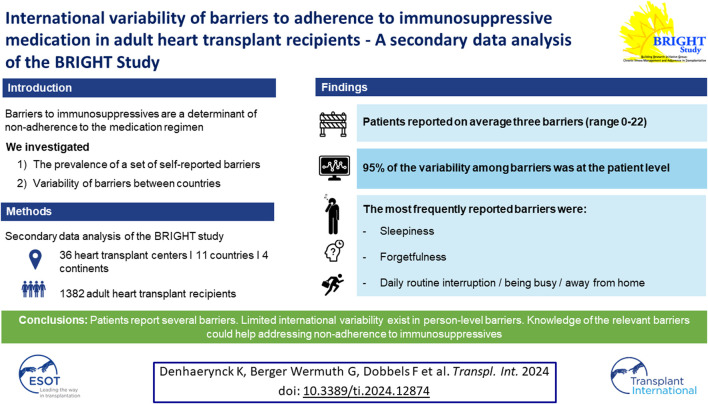

## Introduction

Transplant recipients are required to adhere to a complex medical regimen of lifelong immunosuppressives (IS), supplemented by medications that prevent or treat co-morbidities [1, 2]. Optimal clinical outcome [3–5], and lower costs [6, 7] can be achieved by adhering well to the medication, which implies that the regimen 1) is *initiated* promptly, 2) *implemented* correctly as prescribed and 3) *persistently* continued over time [8]. In order to identify the patients at risk of nonadherence and to know who to support in enhancing their medication taking behavior, knowledge of the determinants of (non)adherence is essential.

Appropriate theoretical models can guide the identification of relevant determinants. The Integrated Model of Behavioral Prediction integrates insights of the most prominent behavioral theories (i.e., the health belief model, theory of reasoned action, theory of planned behavior and the social cognitive theory) [9] into a limited number of determinants of behavior. The model assumes that intentions drive behavior, while their execution can be hindered by a number of non-intentional barriers. Barriers are defined as “a person’s estimation of the level of challenge of social, personal, environmental, and economic obstacles to a specified behavior or their desired goal status on that behavior” [10]. Barriers frequently reported in the transplant literature are forgetfulness [11–21], interruptions to daily routine (e.g., being away from home) [12–17, 19, 22, 23], or having complex medication regimens (e.g., a high number of pills; several intakes per day; medication or dose changes) [14, 20–22, 24]. Barriers to medication taking are an undervalued problem within the transplant population [12, 25], because they are often not recognized strongly associated with non-adherence to immunosuppressives [20, 26], and are predictive of occurrence of acute rejections [27]. So far research has not explored variability in barriers in diverse transplant contexts and healthcare systems.

To overcome the limitations of the hitherto published studies on barriers, which included mostly limited numbers of patients, and limited cultural perspectives and care systems [28–30], the aim of our study was to assess a comprehensive set of barriers to medication adherence using a large multi-center sample of adult heart transplant (HTx) recipients participating in the BRIGHT study [1, 26], to rate the occurrence of the different barriers and assess its variability internationally.

## Materials and Methods

### Design, Setting and Sample

This study is a secondary data analysis of the international multi-center cross-sectional Building research initiative group: chronic illness management and adherence in transplantation (BRIGHT) study [1, 26]. The purpose of BRIGHT was to study variability in health behaviors among HTx recipients internationally, to assess risk-factors for non-adherence at different levels in the healthcare system and to describe and compare practice patterns of chronic illness management. Detailed information on the study methods and procedures has previously been published [1, 26]. In summary, multi-staged sampling of HTx recipients occurred in 11 countries and 36 HTx centers. At least two transplant centers per country were included across four continents: Europe: N = 19 (Belgium, n = 2: France, n = 3; Germany, n = 2; Italy, n = 2; Spain, n = 5; Switzerland, n = 2; UK, n = 3); North America: N = 12 (Canada, n = 4; United States, n = 8); Australia, N = 2; South America, N = 3 (Brazil). Further inclusion criteria for the HTx centers were: a) having performed ≥50 HTx in the 12–60 months prior to inclusion and procuring a formal support letter from the HTx center’s transplant director. HTx recipients were recruited using a proportional random sampling method based on size of transplant center using ISHLT criteria as a basis (i.e., small center: 50–74 HTx/last 5 years; medium center: 75–100 HTx/last 5 years; large center: >100 HTx/last 5 years) [1, 31]. Inclusion criteria of HTx recipients were a) being a ≥18-year-old HTx recipient at inclusion time; b) first single-organ transplant; c) being between one and 5 years post-transplant; and d) managing the taking of medication independently (i.e., without any professional support). All patients gave written informed consent for participation in the study, and approval for the BRIGHT study was obtained by all local ethical committees [1].

### Variables and Measurement

Measurement of variables collected in this study was done using established or investigator-developed instruments by self-report, structured patient interviews as well as medical chart reviews (completed by a nurse or a clinician) [1, 26]. The questionnaires and instruments were pilot tested in diverse settings and translated into the study languages using established protocols.

*Sociodemographic and clinical variables* were age in years, sex, marital status, ethnicity, educational level, employment status, years post-transplant, daily frequency of IS and number of IS per day (see Table 1 for answer categories).

**TABLE 1:** Sample characteristics.

	Overall	BE^a^	FR^a^	DE^a^	IT^a^	ES^a^	CH^a^	GB^a^	CA^a^	US^a^	AU^a^	BR^a^
Gender, N	1,366	74	159	64	111	224	46	98	106	334	51	99
Female, n (%)	375 (27.5)	24 (32.4)	39 (24.5)	15 (23.4)	18 (16.2)	53 (23.7)	14 (30.4)	22 (22.4)	32 (30.2)	106 (31.7)	20 (39.2)	32 (32.3)
Age in years, N	1,349	74	159	65	107	224	46	98	117	331	49	99
mean (SD)	53.2 (±13.2)	52.7 (±12.6)	49.7 (±13)	54.8 (±10.3)	56.5 (±12.5)	55.9 (±11.8)	49.5 (±14.7)	48.8 (±14.8)	54.4 (±13.3)	55.7 (±12.8)	50 (±13.6)	46.3 (±13.3)
Ethnicity, N	1,367	74	158	64	111	221	46	99	115	333	47	99
Caucasian, n (%) Asian African American Hispanic Other	1,176 (86) 26 (1.9) 79 (5.8) 28 (2.1) 58 (4.2)	73 (98.6) 1 (1.4) - - -	142 (89.8) - 2 (1.3) 2 (1.3) 12 (7.6)	64 (100) - - - -	110 (99.1) - 1 (.9) - -	202 (91.3) 1 (.5) - 15 (6.8) 3 (1.4)	42 (91.3) 4 (8.7) - - -	93 (94) 3 (3) - - 3 (3)	103 (89.6) 5 (4.3) 4 (3.5) 1 (.9) 2 (1.7)	250 (75.1) 9 (2.7) 55 (16.5) 9 (2.7) 10 (3)	33 (70.2) 3 (6.4) - - 11 (23.4)	64 (64.6) - 17 (17.2) 1 (1) 17 (17.2)
Marital status, N	1,373	74	158	64	110	224	46	97	116	334	51	99
Single, n (%) Married/living with partner Divorced/separated Widowed	238 (17.3) 948 (69.1) 146 (10.6) 41 (3)	8 (10.8) 56 (75.7) 8 (10.8) 2 (2.7)	35 (22.2) 103 (65.2) 19 (12) 1 (.6)	7 (10.9) 49 (76.6) 6 (9.4) 2 (3.1)	14 (12.7) 83 (75.5) 11 (10) 2 (1.8)	26 (11.6) 156 (69.6) 32 (14.3) 10 (4.5)	8 (17.4) 31 (67.4) 6 (13) 1.(2.1)	26 (26.8) 59 (60.8) 10 (10.3) 2 (2.1)	19 (16.4) 79 (68.1) 10 (8.6) 8 (6.9)	58 (17.4) 234 (70) 29 (8.7) 13 (3.9)	13 (25.5) 34 (66.7) 4 (7.8) -	24 (24.2) 64 (64.7) 11 (11.1) -
Educational level, N	1,370	73	157	64	111	222	46	99	115	336	50	97
Primary school, n (%) Secondary school Post secondary school No scholar education	185 (13.5) 423 (31) 755 (55.1) 7 (.4)	3 (4.1) 42 (57.5) 28 (38.4) -	10 (6.4) 53 (33.8) 94 (59.8) -	7 (10.9) 6 (9.4) 51 (79.7) -	37 (33.3) 51 (45.9) 23 (20.7) -	93 (41.9) 60 (27) 64 (28.8) 5 (2.3)	4 (8.7) 3 (6.5) 39 (84.8) -	- 45 (45.5) 54 (54.4) -	3 (2.6) 33 (28.7) 79 (68.7) -	3 (.9) 70 (20.8) 263 (78.3) -	- 9 (18) 41 (82) -	25 (25.7) 51 (52.6) 19 (19.6) 2 (2.1)
Employment status, N	1,377	74	159	64	111	223	46	99	115	336	51	99
Employed, n (%)	410 (29.8)	18 (24.3)	58 (36.5)	17 (26.6)	33 (29.7)	27 (12.1)	20 (43.5)	37 (37.4)	36 (31.3)	117 (34.8)	25 (49)	22 (22.2)
Years post-transplant, N	1,349	74	159	65	107	224	47	98	110	331	49	99
mean (SD)	3.4 (±1.4)	3.4 (±1.2)	3.7 (±1.4)	3.4 (±1.4)	3.2 (±1.3)	3.6 (±1.4)	3.5 (±1.2)	3.5 (±1.2)	3.7 (±1.5)	3 (±1.3)	4.2 (±1.5)	2.8 (±1.5)
Frequency IS intake/day, N									120	337	51	100
1 time, n (%) 2 times 3 times									1 109 10	3 330 4	- 49 2	- 98 2
Number of IS/day, N									119	335	50	100
Median (Q1-Q3)									9 (6–12)	8 (6–10)	9 (7–11)	7 (6–9)
Barriers^b^												
Number of barriers Mean (SD)	3.01 (±3.98)	3.34 (±3.82)	3.12 (±3.77)	2.69 (±4.51)	1.53 (±2.97)	2.49 (±3.93)	3.61 (±3.88)	3.26 (±3.28)	4.23 (±4.76)	3.20 (±4.04)	5.10 (±4.91)	1.88 (±3.05)
Median (IQR)	1 (0–5)	2 (0–5)	2 (0–5)	1 (0–3)	0 (0–2)	1 (0–3)	2 (0–7)	2 (0–5)	2 (0–8)	2 (0–5)	4 (1–9)	0 (0–3)

^a^ Participating countries: Belgium (BE), France (FR), Germany (DE), Italy (IT), Spain (ES), Switzerland (CH), United Kingdom (GB), Canada (CA), United States of America (US), Australia (AU), Brazil (BR).

^b^ Barrier not present (score: never)/barrier present (score: rarely; sometimes; often; always).

Abbreviation: immunosuppressive medication (IS).

*Barriers to IS adherence* were assessed by written self-report using the 28-item Identifying Medication Adherence Barriers (IMAB) self-report questionnaire [32]. The IMAB was specifically designed for the transplant population, the item generation was based on a systematic review of existing instruments, investigating barriers to medication adherence, published in the chronic illness literature (e.g., forgetfulness; poor health literacy; frequency, number, taste, or shape of IS; costs of IS; see Table 2). To enhance understandability by the patients, IMAB items were slightly adapted by changing the term “anti-rejection medication” into “immunosuppressant medications.” The content validity of IMAB was tested during the Transplant360 project [32], and its internal consistency as part of this study [26].

**TABLE 2:** Prevalence and ranking of barriers overall and top 12 per country.

Barriers^a^	Overall N% (rank)	BE^b^ n% (rank^c^)	FR^b^ n% (rank^c^)	DE^b^ n% (rank^c^)	IT^b^ n% (rank^c^)	ES^b^ n% (rank^c^)	CH^b^ n% (rank^c^)	GB^b^ n% (rank^c^)	CA^b^ n% (rank^c^)	US^b^ n% (rank^c^)	AU^b^ n% (rank^c^)	BR^b^ n% (rank^c^)
Falling asleep/oversleeping	1,379 27.1 (1)	74 27 (3)	158 25.9 (4)	64 21.9 (1)	111 14.4 (1)	224 26.8 (1)	46 28.3 (3)	99 24.2 (5)	117 39.3 (2)	336 31.5 (1)	51 31.4 (6)	99 18.2 (2)
Being away from home	1,380 25.2 (2)	74 23 (4)	158 27.8 (3)	65 15.4 (8)	111 11.7 (3)	223 16.1 (3)	46 30.4 (2)	99 33.3 (2)	117 43.6 (1)	328 27.9 (3)	51 49 (1)	99 11 (6)
Forgetfulness	1,380 24.5 (3)	74 31.1 (2)	158 20.3 (6)	64 17.2 (5)	111 9.9 (4)	224 16.1 (3)	46 23.9 (5)	99 30.3 (4)	117 35.9 (3)	337 31.5 (1)	51 39.2 (2)	99 16.2 (3)
Interruptions to daily routine	1,378 23.6 (4)	32.4 (1)	28.8 (1)	20 (2)	8.1 (6)	17.9 (2)	37 (1)	36.7 (1)	30.8 (5)	22.3 (5)	33.3 (5)	13.1 (5)
Being busy	1,379 22.8 (5)	20.6 (6)	28.5 (2)	17.2 (5)	8.1 (6)	13.4 (7)	28.3 (3)	32.3 (3)	31.6 (4)	26.4 (4)	39.2 (2)	14.1 (4)
Remembering intake of IS	1,378 18.5 (6)	21.6 (5)	22.2 (5)	7.9	12.6 (2)	15.2 (5)	15.2 (10)	20.4 (7)	29.1 (6)	16.9 (8)	27.5 (7)	19.2 (1)
Feeling too sick	1,376 15.7 (7)	17.6 (8)	10.9 (11)	10.9 (9)	5.4	11.7 (9)	8.7	22.2 (6)	22.2 (9)	21.4 (6)	35.3 (4)	5.1
No reminder support	1,376 14.2 (8)	17.6 (8)	17.2 (8)	7.9	6.3 (12)	13.9 (6)	10.9 (12)	17.2 (8)	18.8 (10)	14 (9)	23.5 (9)	10.1 (7)
Holidays or weekend	1,376 13.6 (9)	16.2 (12)	17.3 (7)	17.2 (5)	4.5	11.2 (10)	23.9 (5)	15.3 (9)	23.9 (7)	11	25.5 (8)	3.1
Sticking IS into daily routine	1,377 12.3 (10)	20.3 (7)	11.5 (10)	9.4	7.2 (10)	8.9	10.9 (12)	12.2 (11)	23.9 (7)	11.3 (12)	21.6 (11)	9.1 (8)
Side-effects	1,375 11.5 (11)	17.6 (8)	9.6	17.5 (3)	6.4 (11)	8.9	23.9 (5)	8.2	13.8	11.9 (10)	21.6 (11)	6
Getting IS refill on time	1,378 11.1 (12)	2.7	8.3	6.3	0.9	10.8 (11)	8.7	15.2 (10)	5.1	19.3 (7)	23.5 (9)	7.1 (10)
Inconvenient intake times	1,377 9.9 (13)		14.7 (9)			9.8 (12)	17.4 (8)					
IS intake several times a day	1,377 8.7 (14)						10.9 (12)			11.6 (11)	21.6 (11)	
Going away from home	1,375 8.6 (15)								15.5 (11)			7.1 (10)
Many IS at the same time	1,378 7.8 (16)			10.9 (9)	6.3 (12)				14.5 (12)			7.1 (10)
Difficulties to swallow IS	1,377 7.8 (16)			10.9 (9)	9 (5)		17.4 (8)					7.1 (10)
Intake of IS is noticed by others^d^	1,280 6.8 (18)			10.9 (9)	6.3 (12)							^d^---
Non-understanding of instructions on package	1,373 6.7 (19)	17.6 (8)		17.5 (3)	7.3 (9)	12.9 (8)	11.1 (11)					
Feeling sad or depressed	1,377 6.2 (20)											
Removing IS from package	1,378 6 (21)		10.2 (12)					9.1 (12)				
Bad taste of IS	1,378 4.9 (22)				8.1 (6)		10.9 (12)					8.1 (9)
Costs for IS	1,372 3.6 (23)	1.4	0.6	1.6	0	4	2.2	1	1.7	6.5	9.8	7.1 (10)
Feeling good	1,378 1.3 (24)											
Uncertainty about how to take IS	1,379 1.2 (25)											
No beneficial feeling	1,373 0.9 (26)											
Non-understanding of intake times	1,376 0.8 (27)											
Non-understanding of IS effect	1,377 0.6 (28)											

^a^ Barrier not present (score: never)/barrier present (score: rarely; sometimes; often; always).

^b^ Participating countries: Belgium (BE), France (FR), Germany (DE), Italy (IT), Spain (ES), Switzerland (CH), United Kingdom (GB), Canada (CA), United States of America (US), Australia (AU), Brazil (BR).

^c^ Ranking per country.

^d^ The Brazilian questionnaires did not provide this item.

Abbreviation: immunosuppressive medication (IS).

Patients rated each of the 28 barrier items on a five point scale (never = 1/rarely = 2/sometimes = 3/often = 4/always = 5). Since answer patterns showed a skewed distribution in favor of the lower frequencies, scores were dichotomized into absence of the barrier (never) versus presence of the barriers (rarely, sometimes, often or always). Next to analyzing the barriers individually, we also calculated the total number of barriers per patient.

### Data Analysis

Analyses were of descriptive nature, using the appropriate measures given measurement levels and distributions of the respective variables. Calculation of the intracluster correlation indicated the percentages of variability of the number of barriers per patient, that could be attributed to the different healthcare system levels (i.e., country, center, patient). Analyses were executed in SAS 9.4 (SAS Institute, Cary, NC).

## Results

### Sample Characteristics

**FIGURE 1:**
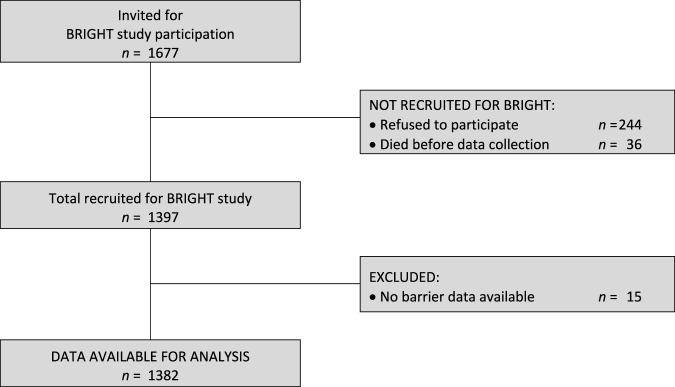
Participant recruitment flow diagram.

Of the 1,397 HTx recipients recruited in BRIGHT study (from an eligible 1,677), 15 (1.1%) did not provide any barrier data and were thus excluded from further analysis for this study (see Figure 1). The remaining 1,382 participants had a mean age of 53.2 years (SD ±13.2) and were, on average, 3.4 years (SD ± 1.4) post-transplant. The majority were male (72.5%), Caucasian (86%), educated at least post-secondary level (55.1%) and married or living with a partner (69.1%). Detailed information on the sample composition is provided in Table 1.

### Number of Barriers Per Patient

The median number of reported barriers was 1 (mean 3.0; SD ± 4.0), with an interquartile range of 5, and ranging from zero (37% patients) to 22 barriers (0.1% of patients). The number of mentioned barriers per participant was diverging too, ranging from an average of 1.5 barriers in Italy to 5.1 barriers in Australia.

### Variability of Barrier Prevalence Between Countries

Calculation of the intracluster correlation showed that 4% of variability of the total number of reported barriers was situated at the level of the country, 1.2% at the level of the centers nested within countries, while the remainder (94.8%) was intra-patient variability.

### Prevalence of Individual Barriers

The prevalence of individual barriers ranged from 0.6% (i.e., non-understanding of IS effects) to 27.1% (i.e., falling asleep/oversleeping). Twelve of the 28 barriers were reported by more than 10% of all participants (Table 2). Five barriers were mentioned by more than 20% of the participants, i.e., falling asleep/oversleeping (27.1%), being away from home (25.2%), forgetfulness (24.5%), interruptions to the daily routine (23.6%) and being busy (22.8%). The percentage of patients who reported at least one of these top five barriers was 50.5%, while 5.3% reported them all at once.

### Differences of Individual Barrier Prevalence Between Countries

Within the different countries, the ranking of individual barriers is similar to the prevalence ranking of barriers overall. The top-12 of barriers differed between countries, with Brazil having the lowest (all 12 barriers <20%) and Australia having the highest prevalence (all 12 barriers >20%). The overall most frequent barrier of falling asleep/oversleeping, appears in the top 6 of all of the countries. The barrier of being away from home, the 2nd most frequently mentioned overall, is equally ranked in the top 6 of barriers in the different countries, except for Germany (where it appeared as rank number 8). Forgetfulness, ranked as 3rd most prevalent barrier overall, is in all countries again in the 6 most prominent barriers. Some barriers were not very prevalent, generally with consistency across countries. One of those lesser reported barriers reflected cost as a barrier, ranked 23rd overall, with a prevalence of 3.6%, the highest being in Australia (9.8%), followed by Brazil (7.1%) and the US (6.5%). Although such a barrier reflects differences in countries’ healthcare systems, the intracluster correlation of this particular barrier only showed 1.2% of the variability to be situated at the country-level (1.2%), the rest being patient-level variability (98.8%).

### Sensitivity Analysis

A sensitivity analysis compared the current ranking of barriers, obtained using frequencies of the dichotomized items (as presented in Table 1) with a ranking based on patients’ mean score, calculated from their responses on the scale from 1 to 5. The Spearman correlation between the two ranking systems was r = .99, indicating that their results were almost identical, thereby validating our dichotomization of item scores.

## Discussion

This study assessed an extensive set of barriers to immunosuppressives intake among a worldwide sample of HTx recipients and found an average of three reported barriers per recipient, with some recipients reporting no barriers, while others up to twenty-two. The five most frequently mentioned barriers were sleeping during a prescribed intake time, being away from home, forgetfulness, interruptions to daily routine and being busy. Half of the sample reported at least one of these five, and one-fifth reported all five to be present simultaneously. These most frequent barriers can be grouped into three themes, which have been mentioned in the literature before:

- *Sleeping* through an intended intake moment, the most frequently mentioned barrier and among the top-3 of barriers in eight of the eleven countries, has been mentioned among renal transplant [33] and chronic heart failure [34] patients, in studies linking daytime sleepiness to poor medication adherence.
- The two barriers *interruptions to daily routine* and being *away from home*, and a third related barrier of *inconvenient intake times*, appeared in more than half of our participating countries and in over ten percent of the participants in six countries. These were previously reported in liver or kidney transplant recipients [14, 16, 17, 23], indicating that stringent intake times of IS can be a challenge at times when the normal schedule is disrupted.
- One of most frequently reported barriers within the transplant literature is *forgetfulness.* [12, 14, 16, 17, 20, 21] Although not ranked first within our sample, this barrier ties together with barriers referring to *difficulties to remember* intake of IS, or of *lacking reminder support,* making this theme one of the important barriers, probably not entirely independent from the previous theme of routine disruptions. As to factors that could explain forgetfulness, is linked with being busy among younger peopl, or to a decline in cognitive abilities among the aged [35]. A person’s personality type also seems to make a difference, since having a more compulsive or anxious personality type support adherence [36]. The meaning of forgetfulness seems to vary somewhat between high and low adherers: qualitative studies have shown that for the former group, forgetting refers to an occasional lapse, whereas for the latter, forgetfulness normalizes a consistent behavioral pattern [37–39].

Despite there being considerable differences between the top-ranked barriers among countries, most of the variability in number of reported barriers was still situated at the recipients level, as shown by the yet small intracluster correlations at the level of countries.

Even the barrier related to the healthcare system – concerning the cost of the IS, had most of its variability situated at the recipient level, despite it being largely determined by policies of healthcare coverage. In European countries, where the healthcare system covers largely the costs of organ transplants and its related expenses (e.g., IS) [40], the prevalence of the cost barrier was expectedly low; while the highest frequencies were recorded in Australia, where almost ten percent of the participants reported cost of IS as a barrier, followed by Brazil and the US. The relatively high frequency of this barrier in Australia and the USA is in line with the findings of a study that showed that their chronically ill patients reported high out-of-pocket costs for healthcare [28], a cause of financial stress [23, 28] and a source of cost-related non-adherence [41]. Unexpectedly, Brazilian recipients also reported a relatively high perception of perceived unaffordability, in spite of the fact that financial coverage for IS also applies to Brazil [40, 42], and that cost-related nonadherence was among the lowest in the Brazilian subsample [41].

### Study Limitations

We investigated barriers to adherence only using the 28 IMAB items, which admittedly primarily focused on patient level barriers. Having the focus primarily on patient level is a limitation in our study. The IMAB could be expanded with additional barriers identified through quantitative and/or qualitative research. Especially barriers at the meso level pointing to barriers in the clinical work flow and organization in transplant centers such as limited time for patient education, not addressing adherence issues during an outpatient clinic visit or lack of trust in or access to healthcare providers might also be considered to be included in a barriers instrument [24]. Another limitation is that although large and with a diverse sample, the Bright study was only cross-sectional, hence, variability and changes in barrier experience over the course of a heart transplantation could not be well documented.

### Implications for Practice and Research

HTx recipients face multiple barriers to adherence to IS. Barriers are proximal determinants of health behaviors and can guide the development of adherence enhancing or remediating interventions. With regard to adherence-enhancing, the advised approach is to first assess adherence and important determinants, such as barriers, in order to identify the patients at risk and deliver a multicomponent behavioral change intervention using shared decision making. Given the impactful nature of poor adherence to IS on clinical outcomes and economic costs [2], health professionals can assess actual and potential barriers a person with a transplant is faced with as this information provides direction in choosing tailored medication adherence interventions. Assessment of barriers in a research study is different from assessing barriers in daily clinical practice. Implementing regular barriers assessment in clinical practice, optimally combined with the assessment of medication adherence as a 5th vital sign (see COMMIT guidelines) [43], calls for careful consideration of context in view of clinical work flow to support the successful implementation in clinical practice (e.g., eHealth tools available for ePROM assessment). Moreover, the information collected needs to enrich clinical decision making. Decision tools integrated in the electronic medical record provide guidance how specific barriers can be linked to adherence interventions. Ribaut et al. have mapped components that can be used [44]. Well-designed interventions also prepare the transplant team and the organization for adherence management [45]. The implementation can be facilitated by dedicated education of transplant clinicians, not only providing the necessary knowledge but primarily with (communication) skills and organizing transplant care based on principles of chronic illness management, so that time and resources are specifically invested in patient’s self management support throughout the transplant journey [2]. An intervention program that successfully implemented all of these principles in a cost-effective way is published by Hooper et al. [46].

As mentioned earlier, barriers instruments can be continuously enriched with multi-level barriers generated from the literature and/or also from clinical observation. The IMAB is a good starting point, however, could be further extended.

### Conclusion

We found limited international variability in primarily person-level barriers in our study. Understanding of barriers in variable contexts guides intervention development to support adherence to the immunosuppressive regimen in real-world settings. Implementation of barriers assessment in daily clinical practice needs specific considerations to guide successful implementation.

## Data Availability

The raw data supporting the conclusions of this article will be made available by the authors, without undue reservation.

## Group Members of The BRIGHT Study Team Consists of

Lut Berben, Institute of Nursing Science, Department Public Health, Faculty of Medicine, University of Basel and UKBB, Basel, Switzerland; Patricia Davidson Johns Hopkins School of Nursing, Baltimore, USA and University of Wollongong Australia, NSW, Australia; Maria G. Crespo-Leiro (Complexo Hospitalario Universitario A Coruña (CHUAC), CIBERCV, INIBIC, Universidade da Coruña (UDC), La Coruña, Spain); Sandra Cupples (U.S. Department of Veterans Affairs, Veterans Health Administration, Washington DC, United States); Paolo De Simone (Azienda Ospedaliero‐Universitaria Pisana, Ospedale Cisanello, Pisa, Italy); Albert Groenewoud (Astellas Pharma Europe Ltd., United Kingdom); Christiane Kugler (Hannover Medical School, Hannover, Germany); Linda Ohler (George Washington University, Washington DC, United States); Johan Van Cleemput (University Hospitals Leuven, Leuven, Belgium); Alain Jean Poncelet (Cliniques Universitaires Saint‐Luc, Brussels, Belgium); Laurent Sebbag (Hôpital Louis Pradel, Lyon, France); Magali Michel (Hôpital Nord Laennec, Nantes, France); Andrée Bernard (Hôpital Universitaire Pitié‐Salpêtrière, Paris, France); Andreas Doesch (University Hospital Heidelberg, Heidelberg, Germany and Asklepios Hospital Bad Salzungen, Bad Salzungen, Germany); Ugolino Livi (University Hospital Udine, Udine, Italy); Luciano Potena (University of Bologna, Bologna, Italy); Vicens Brossa-Loidi (Hospital de Sant Pau, Barcelona, Spain); Javier Segovia-Cubero (Hospital Puerta de Hierro, Madrid, Spain); Luis Almenar-Bonet (Hospital Universitari i Politècnic La Fe de Valencia, Valencia and Hospital Universitari i Politècnic La Fe de Valencia, Valencia, Spain. CIBERCV Hospital Universitari i Politècnic La Fe de Valencia, Valencia, Spain. CIBERCV); Carmen Segura Saint-Gerons (Hospital Univeritario Reina Sofia, Córdoba, Spain); Paul Mohacsi (University Hospital of Bern, Bern, Switzerland); Eva Horvath and Stalder-Ochsner Irene (University Hospital Zurich, Zurich, Switzerland); Cheryl Riotto (Papworth Hospital, Cambridge, United Kingdom); Gareth Parry (Freeman Hospital, Newcastle, United Kingdom); Ashi Firouzi (Royal Brompton and Harefield NHS Foundation Trust, London, United Kingdom); Stella Kozuszko (Toronto General Hospital, Toronto, Canada); Haissam Haddad (University of Ottawa Heart Institute, Ottawa, ON, Canada); Annemarie Kaan (St Paul’s Hospital, Vancouver, BC, Canada); Grant Fisher (London Health Sciences Centre, London, ON, Canada); Tara Miller (Duke University Hospital, Durham, NC, United States); Maureen Flattery (Virginia Commonwealth University Health System, Richmond, VA, United States); Kristin Ludrosky/Nancy Albert (Cleveland Clinic, OH, United States); Bernice Coleman (Cedars-Sinai Medical Center, Los Angeles, CA, United States); Jacqueline Trammell & Flavio Epstein (Kaiser Permanente Santa Clara Medical Center, Santa Clara, CA, United States); Katherine St. Clair, Andrew Kao (St. Luke’s Hospital, Kansas City, MO, United States); Maria Molina (Hospital of the University of Pennsylvania, Philadelphia, PA, United States); Karyn Ryan Canales (Ochsner Medical Center, New Orleans, LA, United States); Samira Scalso de Almeida (Hospital Israelita Albert Einstein, São Paulo and Hospit.al Municipal Vila Santa Catarina - Ministerio da Saude PROAD/-SUS, Sao Paulo, BraziL); Bartira de Aguiar Roza, Paulista School of Nursing, Federal University of Sao Paulo, Sao Paolo, Brazil; ; Andrea Cotait Ayoub (Instituto Dante Pazzanese de Cardiologia, São Paulo, Brazil); Fernanda Barone (Instituto do Coração da Universidade de São Paulo, São Paulo Brazil); Michelle Harkess (St. Vincent’s Hospital, Sydney, Australia); Joanne Maddicks-Law (The Prince Charles Hospital, Brisbane, Australia).
